# The Cassandra Experience: A Mixed Methods Study on the Intragroup Cognitive Dissonance of Italian Expatriates During the First Wave of COVID-19

**DOI:** 10.3389/fpsyg.2021.768346

**Published:** 2021-12-22

**Authors:** Rossella Di Domenico, Davide Cannata, Tiziana Mancini

**Affiliations:** ^1^School of Psychology, National University of Ireland Galway, Galway, Ireland; ^2^Department of Humanities, Social Sciences and Cultural Industries, University of Parma, Parma, Italy

**Keywords:** COVID-19, negative emotions, identity, expatriates, intragroup cognitive dissonance, Italian COVID-19 pandemic

## Abstract

In March 2020, Italy was the first European country to be hit severely by the first wave of coronavirus disease 2019 (COVID-19) and to put in place moderate-high containment measures. 594 Italian expatriates participated in a cross-sectional mixed-methods survey focusing on the period that goes from the beginning of March 2020 to the beginning of April 2020. The survey aimed to describe the experiences of participants when it comes to conflicting beliefs and behavior with the Italian or host country communities in relation to COVID-19, using the Intragroup Cognitive Dissonance (ICD) framework. We explored: (1) COVID-19 risk perception (assessed for themselves, the Italian community, and the host country community); (2) COVID-19 risk meta-perception (participants’ perception of the Italian and host country communities’ risk perception); (3) intensity of emotions (assessed for themselves); (4) national group identification (assessed for themselves in relation to the Italian and host country communities) before and after the first wave of COVID-19 in Italy. An inductive thematic analysis of three open-ended questions allowed an in-depth understanding of the experiences of Italian expatriates. Results describe the ICD of participants with the Italian or host country communities, expressed as a difference between COVID-19 risk-perception and risk meta-perception. ICD predicts that when a dissonance of beliefs and behavior is experienced within an individual’s group, a shift in identification with another more consonant group will happen, if identity enhancing strategies with the dissonant group are unsuccessful. Our findings showed that when the ICD was experienced with the host country community, this was solved through a disidentification strategy and mediated by negative emotions. Identity enhancing strategies with the host country community were unsuccessfully enacted as described by the qualitative answers of participants referring to episodes of racism, ridicule, and to a Cassandra experience: predicting a catastrophic future without being believed. Unexpectedly, participants experiencing the ICD with the Italian community did not enact a disidentification strategy. An increase in virtual contacts, enhanced sense of belonging, a stronger identification baseline, and different features of the two ICDs can be responsible for these results. This study sheds light on the relevance of ICD in natural settings and on international communities, during global crises.

## Introduction

The coronavirus disease 19 (COVID-19) is a highly transmittable and pathogenic viral infection caused by severe acute respiratory syndrome coronavirus 2 (SARS-CoV-2), which first emerged in Wuhan, China at the beginning of December 2019 and rapidly spread all over the world (Shereen et al., 2020). COVID-19 was declared a pandemic on March 11, 2020 (World Health Organization, 2020) and it is considered the first documented coronavirus pandemic and the fifth documented pandemic after the 1918 flu pandemic (Liu et al., 2020). Between February 21 and 23, the number of confirmed COVID-19 cases in Italy sharply raised from 3 to 76 (World Health Organization, 2020). On February 29, the number of confirmed cases in Italy was 888 and Italy was the third most affected country all over the world after China and South Korea (World Health Organization, 2020). Italy remained the most affected country in Europe until April 04 with 119,827 confirmed cases (World Health Organization, 2020). On April 05, the number of confirmed cases in Spain (124,736) overtook that of Italy (124,632) and a surge of cases was registered all over Europe (World Health Organization, 2020). In May 2020, the Americas became the most affected area in the world (World Health Organization, 2020).

Thus, Italy was the most affected country in Europe and the third most affected country worldwide between the end of February 2020 and the end of March 2020 (World Health Organization, 2020). On February 24, Italy applied the first COVID-19 mitigation measures in Northern Italy (see Gobbi et al., 2020), about 15 days before any other country outside the Asia Pacific region. In the weeks ahead, Italy was internationally recognized as the European center of the pandemic. Italian media focused on the dangers of the COVID-19 disease, the importance of preventive measures, and the inability of hospitals to deal with the increasing flood of patients (Cinelli et al., 2020). In the same weeks, the progression of contagion in other countries led Italian scientific authorities to warn about the importance to acting immediately to avoid loss of lives (Saglietto et al., 2020). Italian expatriates might therefore have been exposed to a higher level of alarm than the other people living in the country of expatriation, *via* relevant relationships and media (Christiansen, 2004; Navarrete and Huerta, 2006; McKimmie et al., 2013). This might have led them to experience a contrast between their beliefs about the effects of the pandemic and those of the residents of the host country.

Social psychology could describe this experience of Italian expatriates in terms of cognitive dissonance theory (Festinger, 1957). Cognitive dissonance is described as a mental state in which two cognitive elements are in overt contrast with each other, thus generating an adverse feeling and a consequent need to solve the dissonance (Festinger, 1957). Cognitive dissonance can be influenced by intra-individual or intra-group processes. In the latter case, the behaviors of ingroup members provoke dissonance when they are against the attitudes of someone (Cooper and Stone, 2000; Mckimmie, 2015). Previous studies (Glasford et al., 2008, 2009) defined intragroup cognitive dissonance (ICD) as an adverse mental state determined by an inconsistency between an individual value or belief and the values, beliefs, or behaviors of the other members of the dissonant ingroup. Matz and Wood (2005) identified two drivers that lead individuals to seek consistency between their own beliefs and the ingroup beliefs: a normative driver and an informational driver. The normative driver refers to the desire to maintain harmony with other people in the ingroup. This entails conforming to the social norm, the compendium of the accepted and shared beliefs and attitudes within a group (Hovland and Rosenberg, 1960). The informational driver refers to the desire that personal beliefs and values are validated by the other members of the ingroup. When the consistency between personal beliefs and ingroup beliefs are violated, individuals can experience discomfort and negative emotions (Jetten et al., 2003; Matz and Wood, 2005).

Individuals can also provide strategies to reduce cognitive dissonance. Using a social identity framework (Tajfel, 1978; Tajfel and Turner, 1979), previous studies (Glasford et al., 2008, 2009) found that participants reduced their ICD by decreasing their identification with the dissonant group or engaging in pro-value activism (PVA, e.g., information sharing, persuasion). Similarly, Jetten et al. (2003) observed that participants in an ICD condition experienced a decrease in the intensity of negative emotions when allowed to shift from one group to another or persuade the other ingroup members.

In the current study, the ICD is employed as a theoretical framework to investigate risk perception, group identity, and emotions of Italian expatriates focusing on the period that goes from the beginning of March 2020 to the beginning of April 2020. Due to the early exposure of Italian expatriates to the COVID-19 emergency (*via* Italian media and significant relationships in the home country), the ICD was likely to be experienced by Italian expatriates, as a result of their split identity as Italians and as residents of their host countries. Risk perception is a subjective psychological construct depending on cognitive, emotional, social, cultural, and individual variables (Wildavsky and Karl, 1990; Slovic, 2010; Slovic et al., 1982). Risk perception is an important determinant of the willingness of individuals to embrace health-protective behaviors, according to the protection-motivation theory (Rogers, 1975). Recent findings (Dryhurst et al., 2020) showed that the perception of COVID-19 risk correlated with personal experience with the virus, individualistic and prosocial values, hearing about the virus from friends and family, trust in government, science, and medical professionals, personal knowledge of government strategy, personal and collective efficacy. Social amplification through friends and family was also found to be a significant determinant. Therefore, when the COVID-19 surge of cases in Italy was reported, Italian expatriates who were in contact with their loved ones and exposed to Italian media could have been subject to different attitudes toward COVID-19 than other Italians and the community of their host country. This could lead to ICD with at least one of the two groups and therefore a rise in the intensity of negative emotions (Jetten et al., 2003; Matz and Wood, 2005). The cognitive dissonance might have been solved through disidentification with the most dissonant group and identification with the most consonant group (Glasford et al., 2008, 2009).

The first aim of this study was to describe ICD effects in Italian expatriates by testing the hypotheses that within Italian expatriates the ICDs, i.e., with the Italian community and with the host country community (residents of the host country), correlated to an increase in negative emotions (Hypothesis 1) and that negative emotions were negatively associated with a shift in identification with the Italian or host country community (Hypothesis 2). We also tested the hypothesis that negative emotions partially mediated the relationship between the ICDs of Italian expatriates and the shift in communities identification. Specifically, we assumed that negative emotions activated by the ICDs decreased identification with the dissonant community, and increased identification with the consonant community (Hypothesis 3).

For a better understanding of the real-life ICD phenomenon, we integrated quantitative questions with qualitative questions. Therefore, the second aim of this study was to explore and describe the in-depth personal experience of Italian expatriates with the first wave of COVID-19 in the period examined. This was intended to allow a more detailed picture of the peculiar historical moment explored in this study and possibly inform future research and hypotheses. The qualitative questions depicted the self-reported experience of Italian expatriates concerning risk perception, relationships, and emotions during the COVID-19 emergency in Italy.

## Materials and Methods

A cross-sectional mixed-methods online survey in the Italian language was developed using MS Forms. This was a self-report survey comprehensive of 21 questions of which 18 were close-ended and 3 were open-ended. The questions were allocated into four sections, namely, demographics, perception of COVID-19 risk, sense of belonging and identity, and feelings and emotions. A final section on coping strategies was also present but will be the subject of further analysis following the current study. Consent approval was the only mandatory field in the survey. Data were collected between March 18, 2020 and April 06, 2020.

### Participants

A total of 627 Italian expatriates over the age of 18 and legally able to express their consent, voluntarily participated in the study. To be included in the study participants had to be Italian citizens and reside abroad in any country other than China and South Korea at the time of the study. Eight people opened the link but did not give their consent to participate. Seven participants answered less than 80% of quantitative questions and were therefore excluded from the analysis. For the remaining participants, missing values were computed through the R package “mice” (Van Buuren and Groothuis-Oudshoorn, 2011). We controlled for invariance of answers and multivariate outliers. No response was excluded due to invariance. To investigate multivariate outliers, we calculated Mahalanobis distances. In our dataset 20 cases had a distance score exceeding the critical value (df = 35, χ^2^ = 66.62, α = 0.001). Three participants were excluded as they were living in Italy (2) or in China (1) at the time and two were excluded as no data was available on the country they were currently living in, therefore not fulfilling our inclusion criteria. One participant was excluded from the analysis because of the low quality of qualitative answers.

The remaining 594 participants included 423 women (71.3%), 165 men (27.8%), 3 non-binary (0.5%), and 2 preferred to not answer (0.3%). The mean age was 33.54 (*SD* = 9.13). A breakdown of the host countries of the respondents and their occupational sector is available in a dedicated additional material page on Open Science Framework (OSF^1^). Participants were recruited using two procedures. First, two of the researchers joined different Facebook groups where Italian expatriates or those wishing to relocate, share information and life experiences. Facebook groups were chosen among different cities and countries within and outside the EU. Researchers consistently shared a designated social network post that was subject to the approval of group moderators and which reported the participant inclusion criteria for this study. Second, two researchers independently emailed a designated invitation letter to a cc-blind list of public contacts of Italian expatriates. The list of the Facebook groups joined by each researcher, social network posts, and invitation letters are provided on the Open Science Framework page of the study^1^.

### Measures

#### Risk Perception

The self perception of risk was determined on a single item by asking participants how much they thought that COVID-19 was a threat to their own health on a Likert scale from 1 (very low) to 5 (very high).

Risk perception is defined as how much Italian expatriates think that COVID-19 is an actual threat to the Italian community and the host country community. This was determined by asking participants to estimate on a Likert scale from 1 (very low) to 5 (very high) how much they thought that COVID-19 was a threat to: “friends living in Italy”; “Italian citizens”; “friends living in the host country”; “citizens of the host country.” Risk perception for the Italian community was calculated by averaging the answers “friends living in Italy” and “Italian citizens.” To calculate reliability in all the two-item cases we used the Spearman-Brown split-half reliability formula, as suggested by Eisinga et al. (2013). For the risk perception of the Italian community, ρ = 0.69. Risk perception for the host country community was calculated by averaging the answers “friends living in the host country” and “citizens of the host country” (ρ = 0.80).

#### Risk Meta-Perception

Risk meta-perception is defined as how much Italian expatriates think that the Italian community and the host country community see COVID-19 as a threat. We asked participants to indicate on a Likert scale from 1 (very low) to 5 (very high) how much they thought that different groups considered COVID-19 as a threat. We collected this in relation to “friends living in Italy”; “Italian citizens”; “friends living in the host country”; “citizens of the host country”. The risk meta-perception of the Italian community was calculated by averaging the answers of participants for “friends living in Italy” and “Italian citizens” (ρ = 0.68), while the risk meta-perception of the host country community was calculated by averaging the answers for “friends living in the host country” and “citizens of the host country” (ρ = 0.78).

#### Intragroup Cognitive Dissonance

The ICD was calculated as the expression of the difference between risk perception and risk meta-perception in both Italian and host country communities.

#### Emotions

Participants were asked to indicate the self-reported intensity of a comprehensive range of 20 emotions experienced during the COVID-19 pandemic on a Likert scale from 1 (very low) to 5 (very high). To obtain more meaningful measures, the total number of 20 emotions was reduced by aggregating emotions scores through a Principal Component Analysis in R through the *fa*() function provided by the psych package (Revelle, 2015). The screen plot suggested a 3 factors solution, explaining 42% of the total variance. After varimax rotation Factor 1 was highly loaded to restlessness (0.85), fear (0.82), anxiety (0.75), sense of unsafety (0.71) low mood (0.69), sadness (0.60), frustration (0.57), isolation (0.48), and was labeled “negative emotions”. Factor 2 was highly loaded to joy (0.78), happiness (0.73), serenity (0.63), and fun (0.63) and was labeled “positive emotions”. The third factor showed high loadings to disappointment (0.71) and anger (0.65) and was labeled “oppositive emotions”. Only the negative emotions factor has been used for the models presented in this study. The scale included eight items and reported an alpha reliability value of 0.89.

#### Group Identification

We used single before-after items to ask participants on a Likert scale from 1 (not important) to 5 (very important) the importance given to the identification with the Italian community and with the host country community, before and after the COVID-19 Italian crisis. We calculated the shift in the identification with both groups as the expression of the difference between before and after items. It is important to note that the identification reported before the pandemic was measured retrospectively, therefore participants could only indicate their previous identification as a memory. This represented a weakness of the measure.

#### Qualitative Topics

A set of three open-ended questions was provided at the end of each of the following sections in the questionnaire.

(1) Perception of COVID-19 risk: participants were asked to provide additional information about the perception of COVID-19 risk and any difference between them and the following: “friends living in Italy”; “Italian citizens”; “friends living in the host country”; “citizens of the host country”.
(2) Relationships: participants were asked to provide additional information about any perceived difference in their relationships with the following: “friends living in Italy”; “Italian citizens”; “friends living in the host country”; “citizens of the host country.”
(3) Feelings and emotions: participants were asked to provide additional details regarding the emotional experience during the first phases of the COVID-19 emergency in Italy.

### Analysis

#### Quantitative Data Analysis

**FIGURE 1:**
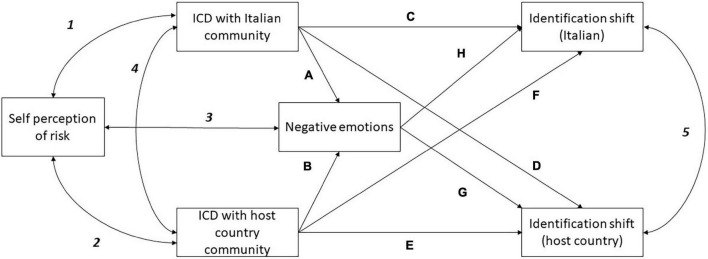
Path model for Hypothesis testing.

Data analysis was conducted using R software (R Core Team, 2019) and the R studio GUI (RStudio Team, 2020). The suite of packages tidyverse^2^ (Wickham et al., 2019) was extensively used for data wrangling and data visualization. A path analysis was used to test our three hypotheses using the *lavaan* package (Rosseel, 2012). For all the other quantitative data analyses the base R package stats was employed. The model tested is presented in Figure 1. In the model, the ICD with both the Italian community (Path A) and with the host country community (path B) positively associated with negative emotions (H1). Negative emotions predicted the shift in identification with both the host country community (path G) and the Italian community (path H; H2). Negative emotions partially mediated the relationship between the ICDs and the identification with both communities. The ICD with the Italian community is predicted to negatively affect the shift in identification with the Italian community (Path C) and positively affect the shift in identification with the host country community (path D). Vice versa, the ICD with the host country community is predicted to positively relate to the shift in the identification with the Italian community (path E) and to negatively affect the shift in the identification with the host country community (path F), as H3 assumed. The following covariances were also controlled: self perception of risk and the ICD with both the Italian community (1) and the host country community (2); self perception of risk and negative emotions (3); the covariance between the ICDs with the Italian and the host country communities (4); and the covariance between identification shifts with the Italian and host country communities (5).

#### Qualitative Data Analysis

For in-depth analysis of qualitative answers collected in this survey, we used an inductive thematic analysis approach (Braun and Clarke, 2006) in which coding and themes were generated without a pre-existing coding scheme. Management and analysis of answers were performed using Nvivo software (QSR International). Analysis began with data immersion, wherein responses were checked and anonymized where necessary. After this, line-by-line coding was conducted. Where the same ideas were identified it was given a code. Codes were subsequently aggregated and themes were identified. The themes were then checked by a second author. The authors that performed and overviewed these analyses have a background in Clinical and Organization Psychology.

## Results

### Descriptive Analysis

Table 1 shows the *M*, *SDs*, and zero-order correlation for each of the variables employed. The difference between the risk perception and risk meta-perception was significant for both the Italian community (ICD with Italian community) and the host country community (ICD with host country community). However, the Italian community risk meta-perception was on average higher than the relative risk perception, [*t*(593) = 4.704, *p* < 0.001, *d* = 0.14], while the risk meta-perception of the host country community was lower than the relative risk perception, *t*(593) = –15.626, *p* < 0.001, *d* = –0.63), showing that Italian expatriates believed that the risk perceived by the Italian community was higher than the actual risk and that the risk perceived by the host country community was lower than the actual risk.

**TABLE 1:** Mean, standard deviation, and zero order correlations of considered measures.

		*M*	*SD*	1	2	3	4	5	6
1	Self perception of risk	2.74	1.02	1					
2	ICD with Italian community	−0.17**	0.89	0.31***	1				
3	ICD with host country community	1.06**	1.00	−0.07	−0.40***				
4	Negative emotions	−0.01	0.93	0.21***	0.15***	0.10*	1		
5	Identification shift (Italian)	0.33**	1.07	−0.01	−0.01	0.19***	0.20***	1	
6	Identification shift (host country)	−0.19**	1.09	0.00	−0.01	−0.13**	−0.12**	0.08	1

*For cognitive dissonances and identification shifts we also report if the means are significantly different from zero.*

*p< 0.05, **p < 0.01, and ***p <0.001.

Also, participants overall felt more identified with the Italian community after rather than prior to the COVID-19 outbreak, [*t*(593) = 7.26 *p* <0.001, d = 0.29) and less identified with the host country community, [*t*(593) = –4.25, *p* <0.001, *d* = 0.13).

### Path Analysis: Model Testing

**FIGURE 2:**
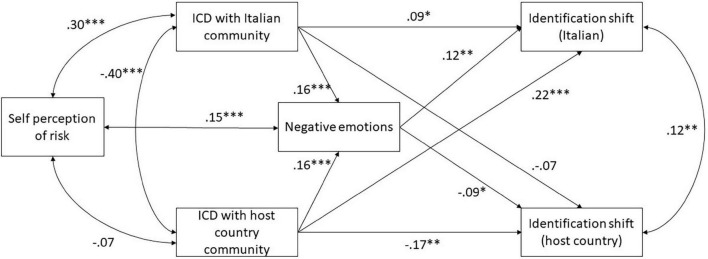
Results of path analysis.

Path analysis with listwise deletion of missing cases showed that the model has an excellent fit with data [χ2 (2, *N* = 598) = 1.019, *p* = 0.413; CFI = 1.00; RMSEA = 0.00)] RMSEA resulted not significant (*p* = 0.751, 90% confidence interval: 0.00–0.067), which is always the case when the Chi-Squared value is smaller than the degree of freedom (Kenny, 2020). The upper and lower limits of the confidence interval are conventionally considered good (Kenny, 2020). All the expected paths were significant except for the one (D) linking the ICD with the Italian community with shifts in identification (Figure 2). As expected, regardless of groups the ICD positively correlated with negative emotions (H1). Negative emotions negatively correlated with the shift in the identification with the host country community as expected (H2), but positively correlated with the shift in the identification with the Italian community. Moreover, as expected by H3, the ICD with the host country community negatively correlated with the shift in identification with that group, and positively correlated with the shift in identification with the Italian community; thus, negative emotions partially mediated the relationship between the ICD with the host country community and the shift in identification with both groups. However, the ICD with the Italian community presented with an unexpected result. It significantly increased the identification with the Italian community rather than decreasing it. Mediation effects of negative emotions were all significant except the one between Italian expatriates and the host country. In the ICD with the Italian community case, negative emotions significantly and totally mediated its association with shift in both identifications. Nevertheless, contrary to our H3, in this case, negative emotions increased identification with the Italian community and decreased identification with the host country community. The full covariance matrix and the details on the estimates for each parameter are published on the OSF page: see text footnote 1.

### Qualitative Results

Five themes were identified during the inductive thematic analysis of the qualitative answers given to the questionnaire. 232 participants answered the first open-ended question (Q1) which related to the perception of COVID-19 related risk. 172 participants answered the second open-ended question (Q2) which related to possible (if any) experienced changes in relationships and contact with other people. 121 participants answered the third open-ended question (Q3) which related to the emotions experienced during the COVID-19 emergency. The total of qualitative answers was 525.

The themes are described below. All the reported quotes generated from the answers given to the questionnaire are translated from Italian to English unless otherwise specified. No location name is reported.

#### (1) Cassandra Experience, Early Measures Undertaking, Racism, and Ridicule

This theme relates to the perception of foreseeing the future without being believed: “*I felt like I wasn’t believed when I said to stay home*” (*Participant 119, Q1*) and includes the perceptions of Italian expatriates in relation to racism and ridicule episodes enacted by the host country community during the COVID-19 crisis in Italy. Comments specifically referred to the Greek myth of Cassandra: *“I was under the impression of being considered by my friends as the Ancient Greeks considered Cassandra, the unheard prophet who only predicted bad news” (Participant 148, Q1)*. Repeatedly Italian expatriates reported having adopted individual COVID-19 containment measures earlier than the host country community: *“Italians (here) started before than locals to limit traveling and social contacts, even before containment measures were put in place” (Participant 106, Q1)*; *“Many Italians started to self-isolate on their own, before the lockdown” (Participant 499, Q1)*, using Italian sources of information: *“I constantly look at Italian news and try to adopt rules suggested by the Italian government” (Participant 118, Q2).*

Episodes of racism were often reported: *“I am from (Italian city), people who know this (here) don’t even say “hello” anymore”; “I felt uncomfortable when talking on the phone in my mother language people started to give me bad looks” (Participant 619, Q2)*. Along with racism, episodes of ridicule were often mentioned: *“I have been discriminated against and people made fun of me” (Participant 215, Q1)*; *“They step back if they see you in a mask and some even laugh at you” (Participant 483, Q1)*. These were associated with a perceived arrogance of the host country community: *“Unable to understand how the virus was going to behave, they arrogantly said that (host country) was better than Italy” (Participant 482, Q1)*. There was a reported perception that the host country community considered COVID-19 exclusively an Italian problem: *“Here it looked like locals pitied Italy without understanding the situation in their own country. In news Italy was depicted as caught up in a social psychosis” (Participant 213, Q1)*, similarly to what happened with Chinese nationals: *“I experienced the same situation with Chinese people…I thought they were exaggerating” (Participant 182, Q1)*.

#### (2) Underestimation of Covid-19 Risk

This theme relates to the perceived underestimation of COVID-19 risk by the host country community: *“My (host country) underestimated the risk since the beginning” (Participant 212, Q1)*; *“Initially largely underestimated. Now increased containing measures have increased perception of the risk, but it is still too low compared to what I think it should be” (Quote originally in English, Participant 27, Q1)*, particularly of its health risks: *“I see too many kids around who don’t realize that they could represent a threat to their (older) relatives” (Participant 42, Q1)*; *“I feel like (host country people) do not engage in safe behaviors despite my fear of the Covid-19 contagion (I have asthma)” (Participant 481, Q2)*. Most participants reported a delay in implementing containment measures in the host country: *“Only a few measures have been taken and with a huge delay” (Participant 625, Q1)* and a slow risk perception: *“Risk perception here does not seem enough, despite the risk evidence” (Participant 95, Q1)*. Some of them reported that at the beginning of the pandemic they personally underestimated the COVID-19 risk: *“At the beginning, I underestimated the situation too, up until Italy went into lockdown” (Participant 218, Q1).* Participants also referred to misinformation: *“(Here) the population has not been informed enough” (Participant 321, Q1)* and unclear communication by the media in the host country: *“I have difficulties to get information about the exact number of cases in my county” (Participant 118, Q3).* Other participants reported that there was inconsistency between population risk perception and the introduction of containment measures by the government of the host countries: *“It was underestimated by people and government at the beginning. Containment measures were then activated in time, but people’s perception of risk is still insufficient” (Participant 594, Q1)*; *“In my host country, despite the government not introducing containment measures, people are being good citizens” (Participant 400, Q1)*.

#### (3) Increase of Contacts

This theme relates to changes in the frequency and/or quality of contacts with friends and/or family determined by the COVID-19 emergency: *“Having lost my job, I managed to be in contact more frequently with friends and family in Italy and with friends and colleagues in my host country” (Participant 39, Q2)*. The increase of contacts was mostly related to family and friends living in Italy: *“We are more in contact, we try to stay close especially with friends and family in Italy” (Participant 133, Q2)*; *“I call my family much more often and I am in contact every day with friends in Italy to check on them” (Participant 54, Q2)*, followed by friends and family living in the host country or other countries: *“I have more contacts with friends and family in Italy but also with friends living in other countries who are facing the same experiences” (Participant 11, Q2)*. Some of the participants reported an increase in contacts with other Italian expatriates living in the host country: *“With this situation, relationships between Italian expatriates consolidated much more, given that we were all in the same situation” (Participant 264, Q2)*. Some participants along with reporting an increase of contacts with people in Italy also reported a decrease of contacts with the host country community: *“My relationship with friends and family in Italy has intensified. In my host country, I had only few relationships, based primarily on casual meetings and some chats, things that do not happen anymore” (Participant 553, Q2).* One participant reported a decrease of contacts with family and friends in Italy caused by anxiety [see also: theme (4)]: *“Lately I can’t call my family and friends very often because I experience anxiety and a bad mood when I call them” (Participant 35, Q2)*. There was a reported change in the content of calls: *“More sensitivity to health topics* (e.g., *Are you ok? How is the isolation going?*)*” (Participant 335, Q2)* and communication methods: *“More relationships via the web” (Participant 103, Q2).*

#### (4) Prevalence of Negative Emotions, Followed by Positive and Mixed Emotions

This theme relates to the emotional experience of participants. Most of the experiences related to negative emotions: *“Lost, nervous and very confused. I don’t know how to react to the whole situation” (Participant 11, Q1)*. Negative emotions were predominantly associated with a preoccupation with loved ones living in Italy: *“preoccupation with their family in Italy unites all Italian expatriates” (Participant 367, Q2)*, preoccupation with the healthcare system of the host country: *“healthcare here is awful” (Participant 35, Q1)*; *“I feel very worried, should anything happen to me here I’d die for sure, they won’t take care of me” (Participant 537, Q1)* and life disruption:*“(the closure of places) has a huge impact on my everyday life” (Participant 551, Q3)*. Losses of relatives were reported: *“I’ve lost two relatives” (Participant 185, Q1)* and change in life decisions: *“I started to question my decision of living abroad” (Participant 198, Q2)*. Fear for economy: *“My preoccupation, being young and healthy, is mainly with economic repercussions” (Participant 530, Q3)*, and critics to Italy were also mentioned: *“Italy is sinking itself not only at the healthcare level but also at the economic level” (Participant 117, Q3)*. Comments related to the perception that economy was preferred over health in the host country: *“(here) they’re more preoccupied of the impact on the economy of the country than of coronavirus risks” (Participant 529, Q2)*. The preoccupation with present and future was also reported: *“preoccupation with present and near-long future” (Participant 16, Q1)*, along with anger at the host countries: *“I felt angry listening to the words of authorities stating that Italy’s containment measures were excessive” (Quote originally in English, Participant 28, Q1)*. Nostalgia was also reported: *“real nostalgia, that makes you appreciate every call or text” (Participant 316, Q2)* and fear of infecting relatives should Italian expatriates return to Italy: *“I fear returning home (to Italy) because this would increase my risk of being infected and therefore infecting (my parents)” (Participant 21, Q1).*

Despite all of this, positive emotions were often mentioned with a predominance of pride for Italy: *“I am proud of how the Italian government acted fast and with strong containment measures” (Participant 28, quote originally in English, Q3)*; *“I am increasingly proud of my country (Italy)” (Participant 215, Q3)* and validation of the host countries COVID-19 response: *“I am in good hands” (Participant 509, Q1)*: *“I feel safe” (Participant 611, Q1)*. Participants also mentioned to have experienced relief when measures were undertaken in their host country, especially when measures were similar to the ones adopted in Italy: *“in a short time containment measures were adopted and similar to the Italian ones: I felt relieved and with a revenge sensation” (Participant 182, Q3)*, and to find comfort in other Italian expatriates: *“My sole source of comfort was my Italian friends in the host country who were able to understand what was happening”* (*Participant 198, Q1*). Mixed emotions were also reported: *“At the moment we are numbed by anger but I am actually starting feeling some beneficial effects” (Participant 10, Q3)*; *“Often contrasting and swinging emotions” (Participant 132, Q3).*

#### (5) Sense of Belonging

This theme relates to a reported sense of common will and belonging to a community: *“This common feeling united people” (Participant 367, Q2)*; *“Very High sense of Belonging” (Participant 5, Q1)*. Most of the time this was referred to the Italian community: *“Increased closeness with Italian friends” (Participant 120, Q2)*. Participants reported feeling closer to the Italian community and expatriates even in the presence of a previous greater or good identification with the host country community: *“I never really felt Italian. I ran away (here) when I was 18, but now I also feel Italian” (Participant 321, Q3); “to me, it has always been important to be part of the host country community. During the emergency, however, I felt stronger my closeness to Italian expatriates” (Participant 137, Q2)*. However, one participant mentioned that the emergency increased their sense of belonging to both communities: *“Emergency increases the sense of belonging to the original community (Italian) and the host country community”(Participant 400, Q2).* There was also a reported sense of belonging without national distinctions: *“This made me discover the feeling of living in a community without national distinctions” (Participant 87, Q2)*.

## Discussion

### Summary of Results

#### Quantitative

Quantitative results of this study showed that the ICD with both the Italian community and host country community determined an increase in the intensity of negative emotions experienced by Italian expatriates. However, the ICD was associated with the hypothesized shift in identification only when experienced with the host country community. Conversely, the ICD with the Italian community produced an unexpected shift in identification in favor of the Italian community rather than the host country community. Negative emotions partially mediated the ICD with the host country community and the shift in identification with both groups. Finally, the identification of Italian expatriates with the Italian community was stronger than with the host country community even before the pandemic.

#### Qualitative

The qualitative results of this study outlined that many Italian expatriates started to perceive COVID-19 as dangerous and implemented the containment measures before the host country community, mostly referring to Italian sources of information and distrusting host country sources [themes (1) and (2)]. This led to a perception of foreseeing the future and feeling “*like Cassandra” (Participant 51, Q1)* referring to the Greek heroine who predicted a catastrophe but was not believed by her fellow citizens [theme (1)]. This perception was echoed by reported experiences of racism, ridicule, and arrogance enacted by the host country communities [theme (1)]. In addition, a reported concern that the host country community was underestimating the COVID-19 health risk was identified [theme (2)]. An increase of contacts, mostly online, was also identified, moreso with the Italian community than with the host country community [theme (3)]. Negative emotions were mostly related to preoccupation with loved ones living in Italy and with the healthcare system of the host country, and sometimes they led to questioning life decisions such as living abroad [theme (4)]. However, fear for the economic consequences of the COVID-19 emergency and criticisms of the Italian response were also mentioned [theme (4)]. Despite this, positive emotions were reported and associated with pride for the Italian response to the emergency. This sentiment was not associated with the host countries. However, the host countries were sometimes associated with a sense of security and good management. Finally, an increased sense of belonging was mentioned and was mostly referred to the Italian community, even alongside with a good identification with the host country community [theme (5)].

### Interpretation of Results

This study demonstrated that ICD effects can be found in real-life situations, but differently from laboratory findings, other uncontrolled variables may also play a role in the ICD effects. For instance, this study found a Cassandra experience, indicating that Italian expatriates perceived that people in the host country did not believe them when they remarked the dangerous health risks of COVID-19, potentially undermining their attempt to maintain good identification with the host country community. Their situation was similar to that of Cassandra, the Trojan heroine who was aware of the danger determined by bringing the wooden horse of Greeks within the city walls, but who was cursed not to be believed (Schapira, 2016). Confirming previous literature, this study shows that when the consistency between personal beliefs and group beliefs is violated, individuals experience ICD, leading to discomfort and negative emotions (Jetten et al., 2003; Matz and Wood, 2005). Furthermore, in line with previous literature (Glasford et al., 2008, 2009), this study showed that decreasing identification with the dissonant group was a chosen strategy among Italian expatriates experiencing the ICD with the host country community. However, this was not the chosen strategy when participants were experiencing the ICD with the Italian community. On the contrary, this study showed an unexpected increase in identification with the Italian community, despite the ICD with this community.

These findings showed that strategies to solve ICD can vary according to the group with which the ICD is being experienced. When the ICD was experienced with the host country community, this was solved through a disidentification strategy. This is in line with previous literature showing that ICD is solved through disidentification with the most dissonant group and identification with the other (Glasford et al., 2008; 2009). Unexpectedly, the ICD with the Italian community did not produce a disidentification with this community. This unexpected result may be due to a major identification with the Italian community, constituting a major source of self-esteem, as postulated by the Social Identity Theory (Tajfel, 1978).

Qualitative results from this study confirm this interpretation. In theme (5) we were able to identify experiences of good baseline identification with the host country community. In theme (1) it was also reported that Italian expatriates were trying to raise awareness in their host country communities of the risks connected with COVID-19, indicating that actions to promote group identification were enacted. However, these actions were not positively valued by members of the host country community. On the contrary, experiences of ridicule and racism were reported, leading to a Cassandra experience [theme (1)]. Cassandra represents a violation of the two factors shown to play a role in group identification: the normative and informational drivers (see Matz and Wood, 2005). On the one hand, Italian expatriates who were experiencing the ICD with the host country community perceived a violation of their desire to maintain harmony with other people of their group (normative driver). On the other hand, the perception of not being believed, as well as the racism and ridiculing actions enacted by the other members of the host country community represented a violation of the desire that personal beliefs and values were validated by the other members of their group (informational driver).

Quantitative analysis showed that Italian expatriates had stronger and more stable national group identification with the Italian community than with the host country community even before the pandemic. Previous literature shows that the identification with new national communities is a complex phenomenon that might involve the amount of time spent in a territory, beliefs of an individual, and their willingness to move or relocate (Faist, 2000; Gustafson, 2006; Castles et al., 2014). We amplify these results showing in our qualitative analysis how experiences of integration are threatened by the ICD with the host country community. A qualitative study published in 2015 (Scotto, 2015) outlines that Italian expatriates show an overall sense of belonging and participation in Italian public affairs and emotional bonding with their homeland. This is echoed by the qualitative results of this study which showed a sense of belonging to the Italian community and interest to maintain contacts with the homeland [themes (3) and (5)]. This might explain why the ICD with the Italian community, although emotionally challenging [theme (4)], did not affect their identification with this national group. Conversely, the identification with the host country community, already being subject to integration processes more recently acquired, might have been weaker and therefore more susceptible to shifts if cognitively and emotionally threatened.

Qualitative findings in this study pointed out that participants were experiencing an overall increment of contacts with family and friends, most of them living in Italy [theme (3)]. This might have contributed to keeping the identification with this community high and undermined the identification with the host country community, with which contacts were decreased. However, an increment of the communication might also be an effect of an already strong baseline identification with the Italian community. In addition, qualitative results showed that positive emotions such as pride for the Italian response to COVID-19 were reported. These might have played a role in the willingness of participants to shift their identification in favor of the Italian community. Despite positive emotions associated with the host country [theme (4)] such as “feeling safe”, most of the comments related to negative emotional experiences concerning the healthcare systems of the host countries [theme (4)]. While this might seem contradictory, based on our analysis the perception of feeling safe was related to the perceived attention to economic consequences by the host country, while the preoccupation with the healthcare system of the host country was related to the perceived underestimation of the COVID-19 risk.

Based on our qualitative results that suggest that the ICD with the host country community bared the perception that the host country community was underestimating the COVID-19 health risk [theme (2)], while the ICD with the Italian community bared mostly preoccupation with economic consequences [theme (4)], we suggest that the ICD with the Italian community was smaller in terms of magnitude, impacts, and consequences in comparison to the ICD with the host country community. The ICD with the Italian community related to the perception that this community was overestimating the COVID-19 risk and was marginal in both quantitative and qualitative data.

COVID-19 containment measures relate to decreasing the proximity of people and increasing personal hygiene and can determine indeed the closure of places in which people congregate, including shops, retail establishments and theatres, causing economic struggle (Pak et al., 2020). Although Italian expatriates might have been affected by the economic and psychological consequences of COVID-19 measures in Italy, not being in direct contact with the Italian community might have limited their effect. Conversely, the health risk of COVID-19 might have had a major effect on Italian expatriates who were experiencing an ICD with the host country community, given their direct contact with this community. In other words, if an ICD with the host country community could represent the exposure to behaviors that were considered unsafe and health-threatening by Italian expatriates (e.g., not following the social distancing measures; not wearing a face covering), this could not be true for the ICD with the Italian community. Recent studies have challenged some aspects of the Cognitive Dissonance Theory, as empirical findings suggested that people can use subtle tactics of negotiations to deactivate the conflict generated by inconsistent ideas (Panagiotou and Kadianaki, 2019). When the sense of urgency was enhanced by concrete personal and community threats, as in the case of the more relaxed behaviors of the host country community toward COVID-19, these more nuanced tactics might have been less likely to be applied, contributing to the disengagement from the host country community. Furthermore, we only explored the differences in the COVID-19 risk perception, but other elements and emotions (particularly, mixed emotions) towards the host or the Italian communities (see Santos et al., 2021) might have played a role in creating an even more contradictory picture. In theme (4) of this study, participants refer to mixed emotions but these were not mentioned in relation to the Italian or host country community but rather to the COVID-19 pandemic in general. It is worth mentioning that a limitation of this study is represented by the way participants reported their identification. While participants reported the identification with the Italian community and the host country community in real-time, they also reported their identification with the two groups before the pandemic as a memory and this is subject to potential distortion.

## Conclusion

This study shed light on the experience of Italian expatriates living through the COVID-19 emergency in another country when Italy was the first European country to be hit severely by the first wave of this pandemic. A combination of quantitative and qualitative methods depicted the complexity of the historical moment in terms of its contribution to social psychology. The COVID-19 emergency has helped to demonstrate the relevance of ICD in natural settings and on a larger social scale. Using this theory, we were able to correctly predict the shift in identification of Italian expatriates in favor of the Italian community when experiencing an ICD with the host country community. We were also able to predict that this was mediated by negative emotions. However, traditionally, ICD predicts similar effects regardless of the direction of the dissonance, while this study found that this was the case only when the ICD was related to the host country community but not when it was related to the Italian community. Italian expatriates experiencing the ICD with the Italian community also reported an increase in identification with the Italian community, despite the ICD. These findings indicate that other variables may play a role in modulating ICD effects.

First, we found that identity enhancing strategies in favor of the host country community were enacted by Italian expatriates resulting in the “Cassandra experience”, the perception of foreseeing the future, enacting actions to raise awareness in the community, but without being believed and therefore discriminated against and ridiculed. Second, we found that the ICD of Italian expatriates with the host country community featured the underestimation of COVID-19 health risks, while the ICD of Italian expatriates with the Italian community featured economic preoccupation. Therefore, the two ICDs bared different connotations. Third, Italian expatriates described an increase of contacts, increase of sense of belonging, and positive emotions toward the Italian community that can be responsible for the identification shift in favor of this community. Fourth, a stronger identification baseline with the Italian community can be responsible for the non-engagement in a disidentification strategy by those experiencing an ICD with the Italian community.

Despite the limitations due to the circumstances of the COVID-19 emergency, having used a mixed-methods survey we were able to add a more nuanced and comprehensive interpretation to our quantitative data, partially overcoming methodological barriers. This study showed that the COVID-19 pandemic had impacts on international communities and shed light on a particular kind of ICD experience, namely the Cassandra experience, that deserves future investigation in natural and/or laboratory settings.

## Data Availability Statement

The datasets presented in this study can be found in online repositories. The names of the repository/repositories and accession number(s) can be found at: https://osf.io/682a3.

## Ethics Statement

Ethical review and approval was not required for the study on human participants in accordance with the local legislation and institutional requirements. The patients/participants provided their written informed consent to participate in this study.

## Author Contributions

RD and DC: conceptualization, methodology, formal analysis, validation, investigation, resources, data curation, writing-original draft, writing-review and editing, visualization, and project administration. TM: conceptualization, resources, formal analysis, writing review and editing, validation, visualization, and supervision. All authors contributed to the article and approved the submitted version.

## Conflict of Interest

The authors declare that the research was conducted in the absence of any commercial or financial relationships that could be construed as a potential conflict of interest.

## Publisher’s Note

All claims expressed in this article are solely those of the authors and do not necessarily represent those of their affiliated organizations, or those of the publisher, the editors and the reviewers. Any product that may be evaluated in this article, or claim that may be made by its manufacturer, is not guaranteed or endorsed by the publisher.

## Footnotes

1
https://osf.io/682a3/

2
https://www.tidyverse.org/
